# Factors Affecting Trailer Thermal Environment Experienced by Market Pigs Transported in the US

**DOI:** 10.3390/ani8110203

**Published:** 2018-11-09

**Authors:** Yijie Xiong, Richard S. Gates, Angela R. Green-Miller

**Affiliations:** Agricultural and Biological Engineering, University of Illinois at Urbana-Champaign, 1304 W. Pennsylvania Ave, Urbana, IL 61801, USA; rsgates@illinois.edu (R.S.G.); angelag@illinois.edu (A.R.G.-M.)

**Keywords:** environment, swine, transport quality, temperature, THI, ventilation, bedding, boarding, misting

## Abstract

**Simple Summary:**

Transport conditions can be a challenge for pigs being transported to market. In this study, 40 trips of commercial market pigs transported from the farms to an abattoir were monitored for thermal conditions including temperature and relative humidity in order to better understand thermal variability within the trailer during transport. Variation in thermal environment inside the pig transport trailer was used as an indicator of ventilation pattern during various weather conditions. During cold weather, the front top and bottom zones were warmer than in the rest of the trailer, indicating less ventilation toward the front of the trailer. Conditions were more uniform throughout the trailer for hot temperatures, indicating sufficient ventilation to limit temperature rise. Misting showed the potential to alleviate high temperatures, but resulted in higher THI conditions. No effect of boarding and bedding combination was observed for spatial distribution of trailer interior temperatures.

**Abstract:**

Extreme weather conditions challenge pig thermoregulation during transport and are addressed by the National Pork Board (NPB) Transport Quality Assurance^®^ (TQA) program that provides guidelines for trailer boarding, bedding, and misting. These guidelines are widely applied, yet very little is known about the microenvironment within the trailer. In this study, TQA guidelines (V4) were evaluated via extensive thermal environment measurements during transport in order to evaluate spatial variability and implications on ventilation pattern. Effects of trailer management strategies including bedding, boarding, and misting were examined and the trailer was monitored for interior temperature rise and THI responses within six separate zones. The trailer thermal environment was not uniformly distributed in the colder trips with the top front and bottom zones were the warmest, indicating these zones had the majority of outlet openings and experienced air with accumulated sensible and latent heat of the pigs. Relatively enhanced thermal environment uniformity was observed during hot trips, suggesting that ventilation patterns and ventilation rate were different for colder vs. warmer weather conditions. Misting applied prior to transport cooled interior air temperature, but also created high THI conditions in some cases. Neither boarding and bedding combinations in the TQA nor boarding position showed impacts on trailer interior temperature rise or spatial distribution of temperature inside the trailer.

## 1. Introduction

Road transport is a critical factor affecting pig welfare in modern commercial pork production and has been reported to increase the number of dead or down (DOD) pigs following transport as the outdoor temperature moves towards extreme hot or cold [1,2,3,4,5,6,7,8,9,10,11]. Despite the understanding of the underlying thermal environmental mechanisms responsible for heat- or cold-stressed pigs, challenging environments are still observed during transport in extreme weather conditions. Limiting the occurrence of poor thermal environments during transport is challenging as current trailer designs provide limited opportunity for modifying internal temperature, humidity, and air velocity [12,13,14,15].

The thermal environment in typical U.S. commercial trailers is not actively controlled, and is affected by many factors, including outdoor temperature, ventilation rate, occupant and bedding sensible and latent heat contributions, pig spatial density, trailer design and boarding management, and transport duration, resulting in conditions that are sometimes undesirable [1,2,3,4,6,7,8,9,10,11,12,13,16,17,18,19]. To address the thermal extremes that may cause distress including dead or down pigs during or after transport, the National Pork Board (NPB) developed and implemented an industry certified program, Transport Quality Assurance^®^ (TQA), to ensure that transported pigs receive a high standard of trailer management to potentially improve trailer thermal environment [20].

The TQA guidelines include recommendations for trailer boarding (the amount of covering of trailer openings) and bedding (presence and depth of a substrate such as wood shavings) that vary with outdoor temperature [14,15,20]. Changes in boarding, in principle, result in changes in net ventilation of the trailer during transport, although variations in boarding patterns (openings toward the front, or the rear, or uniformly along the trailer sides) are not addressed in the TQA. Bedding provides potential insulative effects for the pigs during cold weather and increases footing for the pigs while moving into and out of the trailer, although the likelihood of frozen bedding during extreme cold weather exists [14,15].

Industry implementation of TQA has significantly reduced the number of dead or fatigued pigs at arrival at processing facilities [21]. Past TQA recommendations for bedding, boarding and misting allow for some management practices to vary among producers [20]. For example, under ideal conditions, evaporative cooling has been shown to relieve heat stress conditions and may be achieved by sprinkling pigs and bedding when increased air velocity is provided by fans or during transport [22,23,24,25,26,27]. Alternatively, air cooling by fogging has also shown relief of heat stress conditions by lowering the air temperature. TQA guidelines do not distinguish between these two wetting techniques or provide guidance for how to implement either.

Further exploration of trailer thermal environment under TQA guidelines is merited to better understand the factors contributing to undesirable conditions. Projects commissioned by the NPB to evaluate and revise TQA guidelines found that the guidelines required minimal changes [28,29,30,31,32,33,34]. It was reported by [30] that the highest rate of DOD pigs at arrival occurred when low boarding (<30% coverage) was used for outside temperature < 5 °C. It was found by Kephart et al. and McGlone et al. [31,32] that adding more than 6 bales of bedding did not provide benefits to the pigs, nor did it worsen mortality or morbidity rate during cold weather (<10 °C); however, bedding in excess of 3 bales/trailer during warm weather (>21 °C) showed negative impacts for DOD on arrival. It was found by Kephart et al. and McGlone et al. [33,34] that three methods of sprinkling (on pigs only, on bedding only, or on pigs and bedding) did not have effects for pig measures including pig surface temperature, vocalizations, slips and falls, or transport DOD losses but did show increased stress signs for pigs. The results summarized to date included pig or transport loss measures at the abattoir and averaged or generalized temperatures, rather than comprehensive dynamic measurements of thermal environment during transport. One possible factor affecting the number of DOD pigs is the microenvironment that pigs experience within a trailer. An analysis focused on investigating the variability within the trailer has the potential to identify some conditions that might pose a challenge to a subset of the pigs during transport.

The observational study reported in this paper builds on findings in a companion paper by Xiong et al. [15], where an instrumentation system was designed and implemented into a newly fabricated commercial pig trailer and used throughout the entire study [14,15]. The previous report included 31 trips, with thermal comfort classified as extreme cold, cold, thermoneutral, warm, hot, or extreme hot, based on trailer interior temperatures measured, and did not examine effects of trailer boarding, bedding, or misting on trailer interior temperatures, or explicitly account for the outside temperatures during each trip. The previous paper [15] presented an overview of observations from 31 trips that fully complied with TQA V4, and noted that pigs experienced undesirable thermal conditions for outside temperature below 5 °C or above 27 °C. A Livestock Weather Safety Index in the emergency heat stress category was observed in the trailer when outside temperature exceeded 10 °C. Trailer rear zones most frequently resulted in maximum pig surface temperatures, and middle zones most frequently resulted in minimum pig surface temperatures. Varying boarding levels and distributions showed the potential for altering the ventilation patterns within the trailer and merited further exploration as a technique to increase thermal uniformity throughout the trailer by manipulating the location of fresh air inlets and outlets. This paper provides a more detailed analysis of transport trailer microenvironment to understand spatial variability within the trailer and includes a total of 40 trips. Specifically, this study addresses two objectives:

(1) to assess the spatial variation of the thermal environment in the trailer during 40 commercial transport trips of market-weight pigs under different weather conditions;
(2) to evaluate the effects of trailer management methods including bedding and boarding for cold weather, and misting strategy for hot weather, on trailer interior temperature and temperature and humidity index (THI).

## 2. Materials and Methods

### 2.1. General Information

This paper summarizes results from a multi-year commissioned study that involved three to five monitoring trips every month for over a year, with each monitoring covering a complete transport trip for market-weight pigs (from loading pigs at a pig barn to finish unloading at an abattoir) in the Midwestern U.S. The objective of the commissioned study was to evaluate effects of industry implementation of trailer management (bedding, boarding, and wetting) on trailer interior thermal environment as outlined in the TQA handbook [20]. Therefore, the field measurement was dictated by the outdoor conditions and the specifications of the TQA guidelines. The detailed information of the trailer description, instrumentation of the monitoring system, analysis of pig surface temperature, pig mortality on arrival, effects of trailer bedding depth and boarding percentage on pig skin surface temperature during cold and mild weather conditions are found in the companion paper that characterizes the observations over all weather conditions [15].

### 2.2. Trailer Description and Measurement System Overview

**Figure 1:**
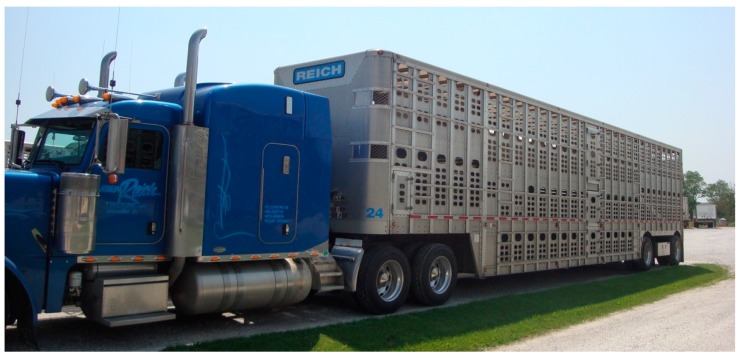
Trailer used for trailer environment monitoring in this field study. The same trailer was instrumented and utilized for all monitoring of trailer interior environment during all commercial market-weight pigs transport throughout this study.

A newly fabricated commercial double-decked pot-belly livestock transport trailer designated for pig transport was used in this study (Figure 1). While this style trailer can be converted to a three-deck configuration for transporting weaned piglets, the trailer in this study was used with only two decks for market pigs throughout the study. The trailer was equipped with three hinged gates on each deck and a loading ramp deployed at the bottom rear. Four 25 × 25 cm nose vents are located in the front corners of the trailer (two to a side, as shown in Figure 1). All nose vents are completely open during hot weather and completely covered during winter. The trailer was divided into six animal zones, numbered from 1 to 3 on the top and 4 to 6 on the bottom deck, from the front to the back. A monitoring system was developed to measure thermal conditions inside each of the six trailer zones. A detailed trailer schematic with zone compartmentalization and monitoring system can be accessed from the companion paper from the same study [15]. The monitoring system in each zone consisted of 14 thermistors (Model 10M5351, Honeywell Parts, Phoenix, AZ, USA), for a total of 84 thermistors in the trailer, to measure pig-level air temperature and one centrally-located temperature and relative humidity (RH) probe (Vaisala INTERCAP HMP60, Vaisala, Vantaa, Finland) per zone to capture the center-zone condition near the ceiling. A weather station was installed outside of the trailer to capture the outside temperature (T_out_) and RH. Data was recorded every minute.

### 2.3. Procedures during a Commercial Pig Transport Trip

During a typical commercial pig transport trip in the Midwestern U.S., the trip generally proceeds in the following segments: (1) arriving at a commercial pig barn and loading market-weight pigs, where variable waiting time at the barn can occur; (2) departing for road transport, and the duration of the transport varies greatly due to distance between scattered barn locations and the abattoir; (3) arriving at the abattoir and unloading the pigs, where waiting time may likely occur due to uncertain processing schedules at the abattoir. During summer conditions, additional cooling procedures may be additional to the trip, based on availability at the barn or abattoir. As for this study, we observed two combinations that can be flexibly available to the trailer operator, including applying misting to the trailer interior at the pig barn, and applying misting at the abattoir, with or without accessing air flows compensated by external fans.

### 2.4. Summary of Field-Monitoring Trips

A total of 40 commercial transport trips for market-weight pigs were successfully monitored with the instrumentation system from May 2012 to February 2013, covering a wide distribution of outdoor conditions, including extreme temperature events [14]. The same trailer was used, and the same driver was responsible for operating and configuring the trailer and managed the animals during all monitoring trips to avoid discrepancy in trailer design, management, and animal handling. All trailer procedures, including misting the interior trailer, bedding and boarding arrangements followed TQA general recommendations. Truck average velocity was approximately the same across all monitoring trips. All trips were conducted with full loading capacity (170–175 market-weight pigs at 127–136 kg each). With a 79.2 m^2^ total trailer floor space, the loading density was 275–300 kg/m^2^.

These 40 trips were categorized into five thermal categories based on average T_out_ recorded during each trip. Table 1 summarizes the thermal categories and the number of trips included in each category. Analyses of trailer management were broken into cold weather analysis that included trips in *Very Cold* and *Cold* categories, and hot weather analysis that included trips in *Mild*, *Warm*, and *Very Hot* categories.

### 2.5. Evaluation of Hot Weather Trips

#### 2.5.1. Misting Procedure

Misting the inside of a stationary trailer and/or access to external fans when available is suggested as an option by the TQA [20] for summer conditions, although the placement and the operating pressure of nozzles are not clearly addressed and can be customized by transport companies. Misting in this study indicated spraying water into the air or onto the back of pigs and/or onto the bedding materials. For the trailer used in this study, 20 misting nozzles (TX-V626, Teejet Technologies, 2 in Zone 1, 6 in Zone 2, 2 in Zone 3, 1 in Zone 4, 6 in Zone 5, and 3 in Zone 6) were located along the middle length on the bottom level, and the right-side length on the top level. While loading pigs at commercial pig barns, two methods of misting were observed based on the water availability, including misting during the process of loading the pigs onto the trailer, or misting briefly after the trailer was fully loaded. Fan banks were located in the abattoir and were available only when the trailer was parked by the fan banks prior to unloading. The duration of misting varied among practices and usually lasted about 5 to 15 min.

#### 2.5.2. Effects of Trailer Management Methods for Hot Weather

Temperature measurements from all of the thermistors in each zone were averaged for the segment during transport (depart from pig barn where pigs were loaded until the arrival at the abattoir). A temperature and humidity index (THI) was computed (Equation (1)) using the center-zone temperature and RH data [14,25]. The average and maximum THI inside the trailer, and the average THI for outside condition during the transport segment were obtained.

(1) $$THI=0.8T_{db}+RH\left(T_{db}-14.4\right)+46.4$$

Four response variables were tested individually to assess the spatial thermal variability within the trailer during transport: (1) average temperature difference between the trailer zones and the outside condition; (2) average THI; (3) maximum THI; and (4) difference between the maximum THI recorded in the trailer and the average THI for outside condition. Each response variable was analyzed for 20 hot weather trips in thermal categories including *Warm*, *Mild*, and *Very Hot* by analysis of variance (ANOVA) for effects of thermal category, trailer zones, misting methods at loading (no misting, misting during loading process or misting after all pigs were loaded), and zone x misting interaction. The analyses were done by PROC MIXED in SAS (version 9.4, [35]). PROC UNIVARIATE was used to verify normality of the dependent variable and accepted at *p* > 0.05. The Tukey–Kramer test for differences of least square means was used to determine significant differences between variable means (*p* < 0.05) due to unequal sample sizes.

#### 2.5.3. Temporal Thermal Profile inside the Trailer for Hot Weather

Center-zone temperatures in the six trailer zones, the outside temperature and center-zone THI conditions were plotted against elapsed time for two representative monitoring trips, including a morning trip in the *Very Hot* category and an afternoon trip in the *Warm* category. Important segments, including arriving at a pig barn, loading pigs onto the trailer, misting inside trailer, road transport, arrival at abattoir, access to fans and misting during waiting, and unloading pigs are identified. The temporal temperature profile was investigated for both hot and cold weather conditions, and the temporal THI profile was conducted for the hot weather conditions only, with one representative sample trip from each data set.

#### 2.5.4. Spatiotemporal Visualization of Variability inside the Trailer for Hot Weather

Temperature distribution patterns on a trailer deck basis (top or bottom) provide visualization of multidimensional temporal variability within the trailer and insight into ventilation patterns during transport. Based on our understanding of the ventilation patterns, cooler regions indicate proximity to an air inlet and hotter regions indicate air outlets in the trailer, except in the case of misting or sprinkling. This is expected from the understanding of pressure distributions on the outside of a moving trailer, with lower pressure inside the trailer that drove air in.

Data from 84 pig-level temperature sensors were linearly interpolated in Matlab^®^ to develop a series of animations that represent the dynamic spatiotemporal profile across the trailer inside [14]. The animations are interpreted as follows: red indicates warmer temperatures and blue indicates cooler temperatures; green circles represent pig-level thermistors and their locations within the trailer; and a colored text box indicates critical events which occurred during the monitoring trip. The animations subjectively describe the ventilation patterns within the trailer, areas receiving benefits from the cooling methods (including misting onto pigs and access to external fans), and the evaporative cooling persistence into the transport segment.

The spatiotemporal visualization of trailer interior temperatures was developed for both hot and cold weather conditions. Representative trips monitored during *Very Hot* (2 trips), *Warm* (1 trip), and *Mild* (1 trip) categories were selected to create the animations. Table A1 (Appendix A) provides the descriptive information for these four trips, with viewable animations available in the Supplemental Materials accompanying this article.

### 2.6. Evaluation of Cold Weather Trips

#### 2.6.1. Boarding and Bedding Procedures

Trailer boarding (covering of trailer openings) was recommended in the TQA for winter conditions. The TQA guidelines outlined 25%, 50%, 75%, and 90% boarding coverage for specific winter outside temperature ranges. Three variations in boarding patterns (uniformly along the trailer sides, boarding gradually more towards rear, and boarding all at the back) are not addressed in the previous TQA guidelines and are evaluated in this study. The industry often applies boarding uniformly along the trailer side.

Bags of conventional kiln-dried pine shavings were used as bedding in this study (0.06 m^3^ each). The use of bedding was characterized by the number of bags placed onto the trailer prior to each monitoring trip and was designated for specific outside temperature ranges: light bedding (1 or 2 bags); medium bedding (3 bags); and heavy bedding (4 to 6 bags). According to the TQA guidelines, the trailer operator had the flexibility to slightly adjust the number of bags of bedding within each designation [20].

#### 2.6.2. Effects of Trailer Management Methods for Cold Weather

Trailer management methods for cold weather investigated were trailer boarding percentage, bedding level, and boarding position. A boarding-bedding combination was created for the analysis, where LM indicates light boarding (25%) and medium bedding (3 bags); MM indicates medium boarding (50%) and medium bedding; and MH indicates medium boarding and heaving bedding (4–6 bags).

The average temperature difference between the trailer zones and the outside condition was analyzed for 16 trips under T_out_ thermal category *Cold* by ANOVA for effects of trailer zones, combination of bedding and boarding percentage, boarding position (as a nesting factor in the boarding and bedding combination, including boarded evenly, more towards the rear, or all at the rear), and zone x boarding position interaction. The analysis was performed by PROC MIXED statement in SAS (version 9.4, [35]). PROC UNIVARIATE was used to verify normality of the dependent variable and accepted at *p* > 0.05. The Tukey–Kramer test for differences of least square means was used to determine significant differences between factor means (*p* < 0.05) due to unequal sample sizes.

For the four trips monitored under the *Very Cold* category, the analysis was simplified due to the lack of combinations of boarding-bedding placement and boarding position in trips monitored, and only the effect of trailer zones on trailer interior temperature rise was analyzed. The boarding-bedding combination and boarding position were not tested. A representative trip in the *Very Cold* category was selected to create the spatiotemporal animation (Table A1 (Appendix A)).

## 3. Results and Discussion

For all 40 trips analyzed, the average duration of a complete trip was 3.5 ± 0.8 h, ranging from a minimum of 0.8 h to a maximum of 4.9 h [14]. The segment during road transport had an average of 2.4 ± 0.8 h, ranging from 0.9 to 4.2 h. A total of four pigs were found DOD for all trips monitored, out of approximately 7000 market-weight pigs transported.

### 3.1. Evaluation of Hot Weather Trips

#### 3.1.1. Effects of Trailer Management Methods for Hot Weather

Table 2 lists descriptive statistics for the 20 transport trips categorized in thermal categories *Mild*, *Warm*, and *Very Hot*. Variables summarized include number of trips, the mean (±standard deviation) of outside temperature, trip duration, and waiting time are provided. The range in average outside temperature varied from 16.7 to 35.3 °C. Mean trip duration averaged 2.1 to 2.7 h, and average waiting times before unloading varied from 4 to 28 min.

Table 3 provides results of effects of trailer misting methods for hot weather management for the corresponding trips included in Table 2. There was no effect of zone on any of the measured variables for these hot weather trips. Results for the main effects (thermal category, trailer zones, and misting methods) are described in the following paragraphs.

**Thermal category.** Results from Table 3 show that the thermal category had a significant effect on the trailer interior temperature rise. For *Mild* and *Warm* categories, trailer interior was warmer than T_out_ but cooler for the *Very Hot* trips, presumably from evaporative cooling of mist water applied prior to transport. When compared to the *Mild* category, average THI was higher for the *Warm* and *Very Hot* categories. THI levels between 78 and 84 are considered dangerous for livestock animals, and THI levels greater than 84 constitute an emergency condition [14,15,25]. We observed dangerous average THI conditions during transport for all trips in the *Very Hot* category, and both dangerous and emergency maximum THI for *Warm* and *Very Hot* categories. The difference between maximum THI recorded in the trailer and the average outside THI was notably different between thermal categories (*p* < 0.001). The difference of maximum THI decreases when the outside temperature became higher.

**Trailer section.** Trailer sections (front: Zones 1 and 4; middle: Zones 2 and 5; and rear: Zones 3 and 6) did not affect the average temperature rise, average THI, or maximum THI. The trailer thermal environment was uniformly hot during each of these trips. The uniform distribution of temperature rises during hot weather trips agree with the results reported by [10], where no difference in temperature among compartments of a double-decked pot belly trailer for weaned piglets was observed for trips with 29 °C average ambient temperature. They did not report the THI conditions in their study.

**Misting.** Misting before the start of trip slightly cooled the trailer for hot weather categories, as measured by average temperature rise in the trailer (*p* < 0.001), maximum THI recorded (*p* < 0.01), and the difference between maximum THI and average outside THI (*p* < 0.001). However, the variation in average temperature rise between no misting, misting during loading, and misting after loaded was less than ±1 °C. Only misting during loading resulted in an average interior temperature cooler than outside. One explanation may be that the average temperature difference over the entire transport segment may mask any relatively shorter-term cooling benefits from misting, which usually only lasted 5–15 min. This is further confirmed by average THI which was also not different between misting at loading categories (*p* > 0.1). A previous study [36] that assessed thermal environment in a goose-neck horse trailer during summer conditions reported that the THI was not uniform in the trailer, and more extreme conditions were found toward the front, suggesting that the front section openings served as outlets. The THI in their trailer was more affected by the ambient condition rather than different trailer positions [36], which matches with our results in which the maximum THI difference was found in the top front zone (ΔTHI_max_ = 9.2), although our study did not note any statistical difference in any THI responses for different zones. The effects of misting after loading, was a higher average temperature rise than misting during loading (*p* < 0.05). The effects of no misting were similar for mean THI to both misting conditions, and a lower maximum THI (*p* < 0.01) than either misting condition, and a lower ΔTHI_max_ (*p* < 0.001) than misting after loading. These results show that one can expect misting during loading to result in similar or reduced interior temperature, similar average THI, and higher maximum THI in the trailer during transport compared to no misting. The use of misting pushed the thermal environment to a dangerous condition for at least some portion of the transport segment, although it did not have a lasting effect.

#### 3.1.2. Temporal Thermal Profile inside Trailer during Hot Weather

**Figure 2:**
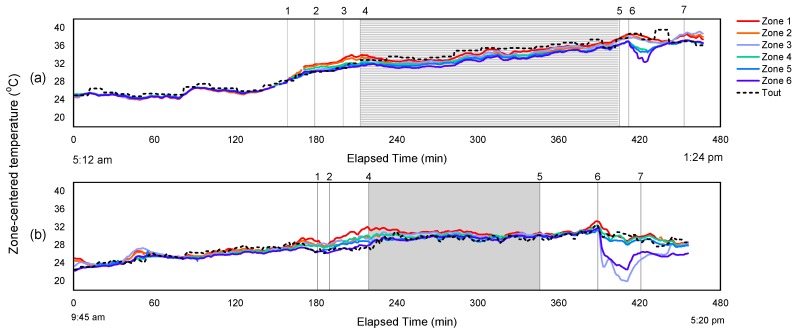
Ceiling-centered temperature profile representing 6 zones inside a pig trailer during: (**a**) a *Very Hot* morning trip; and (**b**) a *Warm* afternoon trip. Zones 1 to 3 represent trailer top deck, and Zones 4 to 6 represent bottom deck. Events occurred during the trip are numbered on the figure as follows (if present): **1.** Arrival at a commercial pig barn; **2.** Loading; **3.** Misting applied inside trailer; **4.** En route; **5.** Arrival at abattoir; **6.** Access to fans and misting during waiting; **7.** Unloading.

**Figure 3:**
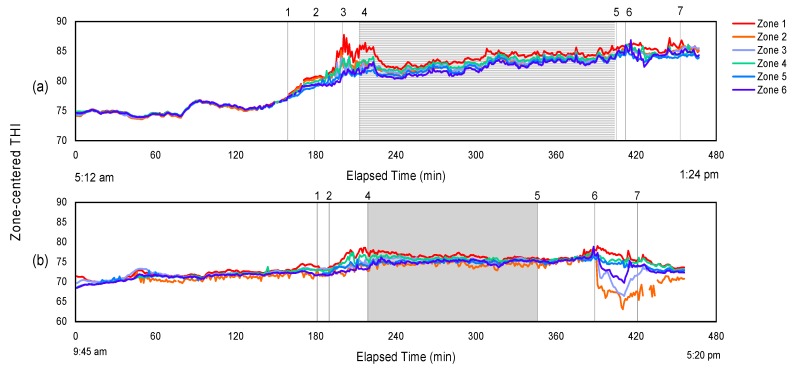
Ceiling-centered THI profile representing 6 zones inside a pig trailer during: (**a**) a *Very Hot* morning trip; and (**b**) a *Warm* afternoon trip. Zones 1 to 3 represent trailer top deck, and Zones 4 to 6 represent bottom deck. Events occurred during the trip are numbered on the figure as follows (if present): **1.** Arrival at a commercial pig barn; **2.** Loading; **3.** Misting applied inside trailer; **4.** En route; **5.** Arrival at abattoir; **6.** Access to fans and misting during waiting; **7.** Unloading.

Obtaining temperature and THI profile of the trailer during the entire course of the transport trip is helpful to understand the nature and to assess the variability of the interior thermal conditions that are encountered by pigs. The spatiotemporal thermal profile was investigated for both hot and cold weather conditions. Figure 2 and Figure 3 show the change of center-zone temperatures and the THI respectively from the six trailer zones with elapsed time from the time the trailer left the home base until the trailer was completely unloaded at the abattoir. The two example trips during hot weather shown here are: (a) a morning trip in the *Very Hot* category that was completed from 5:12 a.m. to 1:24 p.m. and (b) an afternoon trip in the *Warm* category, from 9:45 a.m. to 5:20 p.m. The outside temperature is also plotted on Figure 2, and the trip segment and trailer management were numbered with explanation provided.

Figure 2 and Figure 3a,b demonstrate how trailer interior zone temperatures and THI paralleled the outside temperature during summer conditions, except after arrival at the abattoir when misting with fans was used. In Figure 2, all six zones followed T_out_, with some zones showing more noticeable changes after cooling was applied, and to a lesser extent, prior to the start of road transport as the trailer heated up. The six zone temperatures are nearly identical to the outside temperature when the trailer was empty driving on the road without pigs. Once the trailer stopped for loading, the zone temperatures began to diverge, and then became more uniform again during the transport segment. During loading and cooling, Zones 1 and 2 (top front and middle) were the warmest, although zone effect on average temperature rise was not significant (Table 3). The center-zone THI conditions (Figure 3) paralleled the temperature profile over time (Figure 2), except that the THI substantially increased to approximately 83–85 after misting was applied in the trailer for the trip shown in Figure 2 and Figure 3a, suggesting that the pigs experienced a temporarily dangerous thermal comfort condition for most zones, and an emergency condition for Zone 1. However, this change in thermal comfort condition was not illustrated by the temperature history profile. In the other trip that did not receive misting at loading, the THI in six zones increased when pig loading started, but to a much lesser extent than that with misting applied simultaneously. The visualization of the THI history profile supports our statistical analysis results for the ΔTHI_max_, where an average increase in THI of 4.9 is noted for no misting applied (same case as represented by the trip in Figure 3b), and an increase of 11.1 in THI for misting after loaded (same case as represented by the trip in Figure 3a). In Figure 3a, the discomfort THI condition lasted about 30 min from the onset the misting, and approximately 10–15 mins into the transport segment, but not the entire transport duration. This supports the results that no variation in trailer zones was found for the average THI. For these two representative trips, Zones 1 and 4 (trailer front section) had the most extreme thermal conditions with pigs present for both temperature and the THI. However, this variation between zones were likely masked by the statistical analysis over the entire transport segment.

With regard to misting with fans before unloading used in both trips, substantial non-uniformity in zone temperature and THI was observed. In Figure 2a, interior trailer temperatures in Zones 4–6, representing the trailer bottom deck, rapidly decreased soon after access to misting following fans, resulting in a minimum 4 °C difference between the trailer top and bottom decks; the magnitude of difference was even greater for the trip of Figure 2b, with Zones 5 and 6 showing a temperature reduction of 10 °C cooler than the other zones. In Figure 3a, the THI in the same zones (4–6) decreased similarly to that of the temperatures, but the variation between zones was smaller than that in Figure 2a. In Figure 3b, the THI in both Zones 5 and 6 paralleled the temperature decrease. In addition, the THI in Zone 2 dropped substantially when receiving fans and misting, although the temperature profile in Zone 2 did not show such trend. This further evident that the cooling effect was not uniform in all trailer zones. One explanation for the different zone thermal responses for these two is the uncontrolled trailer parking position, providing different airflow coverage for different parts of the trailer. The uneven benefits from cooling methods were also seen by the pig surface temperature analysis from the same study [14,15]. In a study conducted on broiler transport [37] with external fans at the side of the trailer, simulated ventilation patterns and velocities using a Computational Fluid Dynamics (CFD) model were not uniform for all locations. They concluded that little of the air flow generated by the fans entered the trailer. By adjusting fan heights, they observed a 41% difference in air flow rate in two adjacent top trailer rows, which supports our observation that only the two to three zones on the bottom deck showed any positive effect of fans at the abattoir.

#### 3.1.3. Spatiotemporal Variability inside the Trailer during Hot Weather

**Figure 4:**
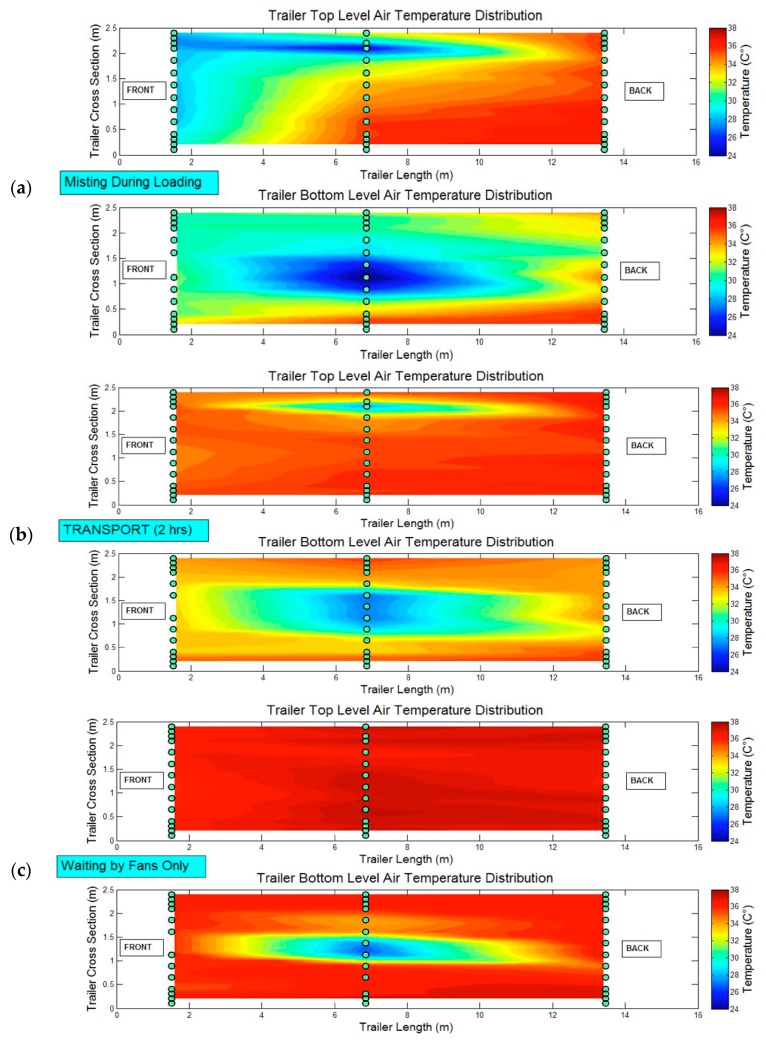
Trailer interior temperature distribution for three events during a hot summer monitoring trip (T_out_ > 32 °C). Lighter color areas represent cooler temperatures inside the trailer, and potentially the cooling effects of the misting lasted into the transport segment. The three events were: (**a**) trailer was stationary, and misting was applied inside onto the pigs and bedding; (**b**) trailer was moving; and (**c**) stationary trailer loaded with pigs at abattoir waiting besides external fans with misting previously applied. The green circles indicate position of air temperature sensors at the pig level.

While the temporal thermal history plots in Figure 2 and Figure 3 illustrate variations in zone temperature and THI over time, the animations of spatial variability in the top and bottom sections of the trailer provide a more detailed view of the thermal variation within and between zones. Figure 4a–c provide animation screenshots for three procedures (misting during loading, as transport is started, and waiting by fans after misting was applied at the abattoir) during a representative *Very Hot* monitoring trip (average T_out_ > 32 °C). The first two procedures, misting during loading and at the start of a trip show substantial spatial variability in both bottom and top decks associated with the misting coverage. During misting, the center zones in the bottom deck were as much as 8 °C cooler than the middle and rear of the top deck. Analysis using only the average temperature of the thermistors, such as depicted in Figure 2, will likely mask this large temperature variation. About 15 min after misting was stopped, the trailer was about to embark on a 2 h journey (Figure 4b), and there were still residual cooling effects noted especially on the lower deck. Upon arrival at the abattoir, misting was applied, and the truck was then moved to the fan bank. The resulting upper-deck temperature is uniform, but extreme (36–38 °C) as was most of the lower deck except in the very center section where some evaporative cooling was still taking place.

These screenshots, and the complete animations provided in the Supplemental Material to this article, illustrate the lack of uniformity in spatial temperature. In the example in Figure 4, this is likely from unequally distributed misting nozzles across the trailer, and the limited effectiveness of the fans at the abattoir.

Misting and fan operation for cooling during hot weather created large temperature variation within the trailer and was not applied uniformly to all pigs, which can be observed in the sample data visualizations (Figure 2, Figure 3 and Figure 4). Misting at loading showed cooling benefits that lasted into the road transport segment, but increased values of THI for at least some portion of the trip. Similar cooling effects that lasted into the road transport segment were observed for misting either during loading or after loaded, but no conclusion can be derived regarding which misting method results in greater temperature depression. The efficacy of misting is affected by the location and direction of the misting nozzles such that the coverage area is optimized, and critically, requires high and uniform velocity distribution to ensure evaporation during transport.

### 3.2. Evaluation of Cold Weather Trips

#### 3.2.1. Effects of Trailer Management Methods for Cold Weather

Results of the effects of trailer boarding and bedding on average temperature difference for *Cold* and *Very Cold* thermal categories is shown in Table 4. Statistical significance between the means for different levels of the analyzed factors is indicated by superscripts in the same row. Boarding position x trailer zone interaction was not significant, thus not discussed.

**Trailer Section.** The trailer zones have a significant effect on the average temperature rise between trailer interior and the outside, indicating the thermal environments between trailer zones were significantly different during transport (*p* < 0.001). For the 16 trips in the *Cold* category, the front section of the trailer (Zones 1 and 4) was warmer than the middle and rear sections, while the middle section (Zones 2 and 5) and rear section (Zones 3 and 6) were not different from one another. For the *Cold* category, the front section was the warmest area of the trailer, while the rear section was the coolest, which indicates the trailer rear section was the air inlet and the front section as the outlet. This result is not consistently the same as that of the hot weather analysis, which reveals that different trailer sections responded to the environment differently between cold weather and hot weather conditions.

**Boarding—Bedding Combination and Boarding Position.** Although Table 4 shows that the main effect of the boarding and bedding combination was significant for the average temperature rise between trailer interior and T_out_, heavier boarding and bedding combination did not show any benefits for increasing trailer zone temperatures. Similarly, for the 16 trips analyzed, none of the boarding positions (as a nesting factor in the boarding—bedding combination) yielded warmer trailer interior temperatures than another. Furthermore, the average temperature rise between trailer interior and the outside was not different based on boarding distribution. More boarding could reduce the air exchange rate by limiting the air inlets and exhaust areas and, hence, the air circulation patterns as well. Since air circulation patterns are affected, air velocity over the animals can also be affected, which in turn theoretically could change convective heat loss. However, our earlier analysis on pig surface temperature as a function of 25% vs. 50% boarding showed no significant difference [15]. Our results agree with [30,31,32], who reported no difference between trailer boarding levels for pig mortality or morbidity for temperature ranging from 5.1 to 23.3 °C; and bedding level did not affect the mortality or morbidity rate at the abattoir.

#### 3.2.2. Temporal Thermal Profile inside Trailer during Cold Weather

**Figure 5:**
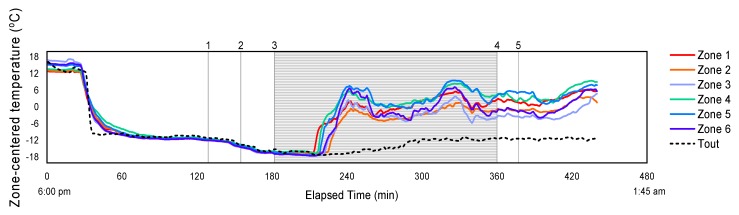
Ceiling-centered temperature profile representing 6 zones inside a pig trailer during a *Very Cold* evening trip. Zones 1 to 3 representing trailer top deck, and Zones 4 to 6 representing bottom deck. Events occurred during the trip are numbered on the figure as follows: **1.** Arrival at a commercial pig barn; **2.** Loading; **3.** En route; **4.** Arrival at abattoir; **5.** Unloading.

Figure 5 show the change of center-zone temperatures from the six trailer zones with elapsed time for a complete transport trip in the *Very Cold* category that was conducted during the evening, from 6:00 p.m. to 1:45 a.m. the next day.

For this winter monitoring trip, variations of up to 8 °C between zone temperatures were observed. The temperature rise added by the pigs’ heat production was more obvious than that of the summer trips, reaching an 18 °C temperature difference on average between trailer interior and T_out_ which was about −14 to −12 °C for most of the trip. This trend was similar to that found by [10,11], where a magnitude of 15–20 °C temperature rise in the trailer was noted for winter trips conducted for weaned piglets transport in Illinois and Iowa. Trailer temperatures during the initial part of the road trip were similar to outside, until about 40 min into the trip when the trailer began to warm up. Zones 3 and 6 (rear of trailer) were consistently colder after the end of the trip, but during transport no clear trends in differences between trailer zones or trailer decks can be seen in this plot, although the front was warmer on average for all cold trips (*p* < 0.05). However, the maximum difference between zone temperatures was 12 to 15 °C. Reasons for this large variability are not clear, but it is likely the amount and placement of boarding affected the relative position of inlets and outlets for the trailer.

### 3.3. Ventilation Implications

Our analysis for cold weather conditions, based on variation in zone temperature rise, showed that the trailer front section was consistently the warmest, while no difference was noted for other zones. For warmer weather trips, no significant difference in the trailer interior temperature rise, average and maximum THI, or maximum THI difference was found between different trailer zones, which indicates a uniform spatial thermal environment. Despite the lack of statistical significance in average temperature rise and THI, our visualized sample data sets observed the most extreme conditions in the front zone for a *Very Hot* and a *Warm* trip. Our results are substantially in agreement with [10], where thermal environment and ventilation patterns were studied for a double-decked pot belly trailer transporting weaned piglets. They reported that the top front section was consistently the warmest location on the trailer for multiple cold weather trips (average ambient temperature of 2 °C, equivalent to the *Cold* category in this study), while the front–middle compartments on both decks, and the top rear compartment, were found to be the warmest locations for trips with average ambient temperature 16 °C (equivalent to the *Mild* category in this study). The middle compartments of both decks were coolest for both ambient temperatures studied. However, no difference in compartment temperature was noted for trips with a higher average ambient temperature (29 °C) which fits the *Warm* category in this study.

Variability of the thermal environment in the trailer can be a useful tool for indication of potential ventilation patterns in a moving trailer. In colder conditions, cooler zone temperatures indicate where air is entering the trailer, and warmer temperatures indicate locations with air outlets and likely less air velocity [10,11,16,38,39]. Previous livestock trailer ventilation studies demonstrated that air inlets are typically at the rear and air outlets toward the front [3,16,38,39,40,41]. Our results agree with this situation for colder weather conditions; the front of the trailer was consistently the warmest area while cooler temperatures were recorded toward the middle to rear sections, suggesting a general air flow from rear to front. During winter trips, the front nose vents were completely covered in this study, much of the side openings were boarded, and only a portion of the rear section could serve as an air inlet. The cold air heats while moving inside the trailer due to sensible energy contributions from the pigs. A greater temperature increase in one area can indicate either less ventilation in that area, or a sufficiently low ventilation rate to allow for sensible heating. In this study, our results indicate a ventilation “dead-spot” at the front during winter, suggesting a relatively low overall ventilation exchange rate, and which resulted in non-uniform thermal distribution. These non-uniformly distributed ventilation patterns are acknowledged by research with multiple livestock animals [10,11,16,37,38,39]: Purswell et al. [16] reported the air exchange rates were notably different across different locations on a slant-load horse trailer, and varied with road speed; Harmon et al. and Zhao et al. [10,11] simulated air flow rate using a 1/7th scale trailer model, and reported a range of 3.4 to 6.9 ms^−1^ air velocity can be expected in a double-decked pot-belly pig trailer; Heymsfield et al. [37] reported different ventilation patterns and rates across cages in a straight-deck broiler transport trailer.

For warm-weather trips, the trailer temperature distribution was relatively uniform in this study, suggesting a different ventilation pattern or rate, as side and front openings are opened completely and the ventilation rate becomes sufficiently high to minimize temperature variation. The trailer nose vents located at the front corners were completely closed during winter but opened during summer (as can be seen from Figure 1, in which the four nose vents are all open; the left corner of trailer front board had two identical vent openings as shown on the picture). During hot weather, it is likely that the fresh air entered into the rear sections of trailer and exited towards the front. With trailer sides completely open and front nose vents uncovered, the lack of temperature or THI rise was the result of sufficient opening areas and the pressure gradient to induce a high ventilation rate, limiting the temperature rise observed during winter.

The varying ventilation patterns or rates can be attributed to many factors: during different seasons, trailer speed, vent openings, and the presence of the rear door panel significantly affected the air exchange rates in a horse trailer [16,38]; front trailer vents notably affected the direction but not the speed of air flows [10]; and interior pen partitions reduced air velocity (hence, air flow) by about 50% [10,11]. Given the relatively solid floors on each deck, the top and bottom decks are effectively independent of each other, with airflow through them dictated by size and location of potential inlets and outlets. A few previous studies supported the possibility that different configurations of side opening covering and rear panel design may also affect the ventilation pattern in the trailer [10,15,16,38]. Our results show that heavier boarding did not appear to reduce ventilation rate, as indicated by a greater average temperature difference for the lightest boarding scenario (Table 4), but the position of boarding likely altered the location of air inlets and exhausts along the sides of the trailer. When placing boarding equally versus distributing it more toward the rear during cold weather, no potential for increasing trailer temperature was observed. These explanations cannot be directly verified because air flow direction and velocity inside the trailer were not directly measured in this study.

## 4. Conclusions

Variation in the six zones within a commercial transport trailer over the course of a year were tested for lack of uniformity against different factors depending on outdoor weather conditions. For 20 trips conducted during *Mild*, *Warm* and *Very Hot* conditions, no significant variation was observed in temperature rise or average THI between the six trailer zones. Ventilation through the trailer was sufficient to limit temperature rise due to sensible and latent heat contributions by the pigs. Misting during loading the trailer with pigs showed modest benefit to cool the trailer interior temperature to less than outside temperature, however, misting did not improve the average THI and created short term heat stress conditions as measured by maximum THI in the trailer, pushing the thermal environment to a potentially dangerous condition for at least some portion of the transport segment. After transport, fans with or without misting reduced temperature rise in some zones of the trailer but the benefits were not uniform.

For 20 *Cold* and *Very Cold* weather trips, the trailer interior thermal environment was not uniform, with the front top and bottom zones being the warmest, indicating less ventilation in these areas, which is in line with results from animal transport assessments for pigs and other species. Boarding/bedding combinations changed ventilation rates but was generally inconsistent with respect to variation in temperature, and boarding position had no measurable effect on ventilation pattern or rate. Unlike the warmer weather situation with full ventilation, a partially boarded trailer reduced the ventilation rate and resulted in a temperature gradient from the rear towards the front, conforming to the air inlet and outlet locations, respectively. The potential implications of reduced ventilation would be a susceptibility to poorer air quality and potentially challenging thermal environment for the pigs, which have significant consequences for pig welfare during cold weather transport.

## Figures and Tables

**Table 1:** Summary of field-monitoring trips completed with thermal categories based on average outside air temperature range recorded during each trip.

Evaluation	Thermal Category	T_out_ Range (°C)	Number of Trips
Cold Weather Analysis	*Very Cold*	<−12	4 *
	*Cold*	−12 to 9	16
	Total cold weather trips		20
Hot Weather Analysis	*Mild*	10 to 26	8
	*Warm*	27 to 32	7
	*Very Hot*	>32	5
	Total hot weather trips		20

* For the *Very Cold* category, one trip experienced thermistor failures and was excluded from this table and analysis involving pig-level air temperatures.

**Table 2:** Descriptive statistics for 20 monitored transport trips categorized as thermal categories *Mild*, *Warm*, and *Very Hot*.

Thermal Category	Misting at Loading	Trips (N)	Total Trips (N)	Mean T_out_ (°C)	Mean Transport Duration (h)	Waiting Time before Unloading (min)		
						with Fans	with Fans and Misting	No Cooling
*Mild*	None	8	8	16.7 ± 4.2	2.1 ± 0.6	18 ± 36	N/A ^1^	28 ± 19
*Warm*	None	1	7	27.0 ± 4.2	2.3 ± 0.5	15 ± 25	4 ± 10	17 ± 16
	During	3						
	After	3						
*Very Hot*	During	2	5	35.3 ± 1.9	2.7 ± 0.7	9 ± 12	10 ± 16	10 ± 5
	After	3						

^1^ N/A: there were no instances of this combination occurring.

**Table 3:** Evaluation of the effects of thermal category, trailer zones, and misting methods for 20 monitored transport trips categorized as *Mild*, *Warm*, and *Very Hot*. The response variables analyzed included average temperature difference between trailer interior and the outside temperature, average and the maximum temperature and humidity index (THI), and the difference between maximum THI and the average outside THI condition.

Response Variable Analyzed	Thermal Category					Trailer Section (Zones) ^1^								Misting at Loading				
	Sig. ^2^	*Mild*	*Warm*	*Very Hot*	SEM	Sig. ^2^	Front		Middle		Rear		SEM	Sig. ^2^	None	During	After	SEM
		(8) ^3^	(7) ^3^	(5) ^3^			1	4	2	5	3	6			(9) ^3^	(5) ^3^	(6) ^3^	
**Average temperature difference between trailer interior and T_out_ (°C) ^4^**	**	1.1 ^A^	0.5 ^B^	−1.1 ^C^	0.5	NS	0.4	0.1	0.1	−0.2	0.4	0.1	1.1	***	0.9 ^A^	−1.0 ^B^	0.5 ^A^	0.5
**Average THI ^5^**	***	65 ^A^	75 ^B^	84 ^C^	0.8	NS	75	75	74	74	74	74	4.4	NS	75	75	74	1.4
**Maximum THI ^6^**	***	73 ^A^	80 ^B^	87 ^C^	0.9	NS	81	81	79	80	80	81	4.6	**	77 ^A^	82 ^B^	82 ^B^	1.2
**Maximum THI difference ^7^**	***	12.2 ^A^	7.4 ^B^	4.9 ^C^	1.1	NS	9.2	8.6	7.2	7.4	7.6	8.6	2.2	***	4.9 ^A^	8.4 ^A^	11.1 ^B^	1.1

^1^ Trailer zones are designated as: zone 1—top front; zone 4—bottom front; zone 2—top middle; zone 5—bottom middle; zone 3—top rear; and zone 6—bottom rear. ^2^ Sig. indicates level of significance for the main effect, where ** indicates a *p*-value < 0.01, *** indicates a *p*-value < 0.001, and NS is not significant (*p* > 0.05). ^3^ Numbers in the parenthesis indicate the number of trips completed and analyzed for this combination. ^4^ Numbers indicate the least square means for the average temperature difference between trailer interior and the outside temperature for the during transport segment. ^5^ Numbers indicate the least square means for the average THI for the during transport segment. ^6^ Numbers indicate the least square means for the maximum THI for the during transport segment. ^7^ Numbers indicate the least square means for the difference between the maximum THI documented for inside the trailer and the average THI for the outside condition for the during transport segment. ^A–C^ Different superscripts within a row under the analyzed factor indicate the means differ significantly (*p* < 0.05) using the Tukey–Kramer test for differences of least squares means.

**Table 4:** Evaluation of the effects of trailer zones, bedding and boarding combination, and boarding position for 20 monitored transport trips categorized as thermal categories *Cold* and *Very Cold*.

Thermal Category	Trips (N)	Mean T_out_ (°C)	Mean Duration (h)	Average Temperature Difference between Trailer Interior and T_out_ (°C) ^1^																			
				Trailer Section (Zones) ^2^								Boarding—Bedding					Boarding Position						
				Sig. ^3^	Front		Middle		Rear		SEM	Sig. ^3^	LM	MM	MH	SEM	Sig. ^3^	LM		MM	MH		SEM
					1	4	2	5	3	6			(6) ^4^	(5) ^4^	(5) ^4^			Even (3) ^4^	Rear (3) ^4^	Even (5) ^4^	Rear (2) ^4^	Back (3) ^4^	
Cold	16	7.0 ± 2.4	2.5 ± 0.8	***	3.9 ^AB^	5.0 ^A^	2.1 ^C^	2.3 ^BC^	1.8 ^C^	2.1 ^C^	0.9	**	3.6 ^A^	2.6 ^B^	2.5 ^B^	0.6	NS	4.0	3.2	2.6	2.3	2.6	0.6
Very Cold	4	−10.6 ± 3.4	2.3 ± 1.0	**	13.6 ^A^	13.6 ^A^	8.1 ^BC^	14.4 ^A^	4.5 ^C^	11.4 ^AB^	3.4	NA ^5^					NA ^5^						

^1^ Numbers are the least square means for *Cold* category and means for the *Very Cold* category. ^2^ Trailer zones are designated as: zone 1—top front; zone 4—bottom front; zone 2—top middle; zone 5—bottom middle; zone 3—top rear; and zone 6—bottom rear. ^3^ Sig. indicates level of significance for the main effect, where ** indicates a *p*-value < 0.01, *** indicates a *p*-value < 0.001, and NS is not significant (*p* > 0.05). ^4^ Numbers in the parentheses indicate the number of monitoring trips completed and analyzed for this combination. ^5^ NA indicates insufficient observations across levels of main effect for testing. ^A–C^ Different superscripts within a row under the analyzed factor indicate the means differ significantly (*p* < 0.05) from the Tukey–Kramer test for differences of least squares means.

## Supplementary Materials

The following are available online at http://www.mdpi.com/2076-2615/8/11/203/s1, Animation A1: Animation of a complete commercial pig transport trip during very hot weather condition (1). Animation A2: Animation of a complete commercial pig transport trip during very hot weather condition (2). Animation A3: Animation of a complete commercial pig transport trip during warm weather condition. Animation A4: Animation of a complete commercial pig transport trip during mild weather condition. Animation A5: Animation of a complete commercial pig transport trip during very cold weather condition.

## Appendix A

The descriptive information of the animations is interpolated from pig-level air temperature sensors for five representative trips that covered extreme outside thermal categories and encountered representative combinations of critical trailer management. The contents were discussed in Materials and Methods, Results, and Discussion. Complete animations can be accessed from the Supplementary Materials.

**Table A1:** Descriptive information of the five animations with reference to pages 6, 11–13.

	Animation 1	Animation 2	Animation 3	Animation 4	Animation 5
Outside Temperature	>32 °C	>32 °C	27–32 °C	10–20 °C	<−12 °C
Thermal Category	*Very Hot*	*Very Hot*	*Warm*	*Mild*	*Very Cold*
Trailer Management	Misting	Misting	None	None	90% boarding
Cooling at the Abattoir	Fans & misting	None	Fans & misting	None	None
Transport Duration (h)	3.2	2.0	2.1	1.0	2.5

## References

[1] Ritter M.J., Ellis M., Bowman R., Brinkmann J., Curtis S.E., DeDecker J.M., Mendoza O., Murphy C.M., Orellana D.G., Peterson B.A. (2008). Effects of season and distance moved during loading on transport losses of market-weight pigs in two commercially available types of trailer. J. Anim. Sci..

[2] Ritter M.J., Ellis M., Anderson D.B., Curtis S.E., Keffaber K.K., Killefer J., McKeith F.K., Murphy C.M., Peterson B.A. (2009). Effects of multiple concurrent stressors on rectal temperature, blood acid-base status, and longissimus muscle glycolytic potential in market-weight pigs. J. Anim. Sci..

[3] Ellis M., Wang X., Funk T., Wolter B., Murphy C., Lenkaitis A., Sun Y., Pilcher C. Impact of trailer design factors on conditions during transport. Proceedings of the 2010 Allen D. Leman Swine Conference.

[4] Sutherland M., McDonald A., McGlone J. (2009). Effects of variations in the environment, length of journey and type of trailer on the mortality and morbidity of pigs being transported to slaughter. Vet. Rec..

[5] McGlone J.J., Johnson A.K., Sapkota A., Kephart R.K. (2014). Transport of Market Pigs: Improvements in Welfare and Economics. Livest. Handl. Transp. Theor. Appl..

[6] Peterson E., Remmenga M., Hagerman A.D., Akkina J.E. (2017). Use of Temperature, humidity, and slaughter condemnation data to predict increases in transport losses in three classes of swine and resulting foregone revenue. Front. Vet. Sci..

[7] Brown J.A., Samarakone T.S., Crowe T., Bergeron R., Widowski T., Correa J.A., Faucitano L., Torrey S., Gonyou H.W. (2011). Temperature and humidity conditions in trucks transporting pigs in two seasons in eastern and western Canada. Trans. ASABE.

[8] Schwartzkopf-Genswein K.S., Faucitano L., Dadgar S., Shand P., González L.A., Crowe T.G. (2012). Road transport of cattle, swine and poultry in North America and its impact on animal welfare, carcass and meat quality: A review. Meat Sci..

[9] Voslarova E., Vecerek V., Passantino A., Chloupek P., Bedanova I. (2017). Transport losses in finisher pigs: Impact of transport distance and season of the year. Asian-Australas. J. Anim. Sci..

[10] Harmon J.D., Hoff S.J., Baas T.J., Zhao Y., Xin H., Follet L.R. (2017). Evaluation of conditions during weaned pig transport. Appl. Eng. Agric..

[11] Zhao Y., Xin H., Harmon J.D., Baas T.J. (2016). Mortality Rate of Weaned and Feeder Pigs as Affected by Ground Transportation Conditions. Trans. ASABE.

[12] Correa J.A., Gonyou H.W., Torrey S., Widowski T., Bergeron R., Crowe T., Laforest J.P., Faucitano L. (2014). Welfare of pigs being transported over long distances using a pot-belly trailer during winter and summer. Animals.

[13] Conte S., Faucitano L., Bergeron R., Torrey H.V., Gonyou H.W., Crowe T., Tamminga E.T., Widwoski T.M. (2015). Effects of season, truck type, and location within truck on gastrointestinal tract temperature of market-weight pigs during transport. J. Anim. Sci..

[14] Xiong Y. (2013). Evaluation of Trailer Thermal Environment during Commercial Swine Transport. Master’s Thesis.

[15] Xiong Y., Green A., Gates R.S. (2015). Characteristics of Trailer Thermal Environment during Commercial Swine Transport Managed under U.S. Industry Guidelines. Animals.

[16] Purswell J.L., Gates R.S., Lawrence L.M., Jacob J.D., Stombaugh T.S., Coleman R.J. (2006). Air Exchange Rate in a Horse Trailer during Road Transport. Trans. ASABE.

[17] Warriss P. (1998). Choosing appropriate space allowances for slaughter pigs transported by road: A review. Vet. Rec..

[18] Scheeren M.B., Gonyou H.W., Brown J., Weschenfelder A.V., Faucitano L. (2014). Effects of transport time and location within truck on skin bruises and meat quality of market weight pigs in two seasons. Can. J. Anim. Sci..

[19] Pérez M.P., Palacio J., Santolaria M.P., Aceña M.C., Chacón G., Gascón M., Calvo J.H., Zaragoza P., Beltran J.A., Garcia-Belenguer S. (2002). Effect of transport time on welfare and meat quality in pigs. Meat Sci..

[20] National Pork Board (2008). Transport Quality Assurance Handbook, Version 4.

[21] National Pork Board (2009). Quick Facts: The Pork Industry at A Glance.

[22] Fox J., Widowski T., Torrey S., Nannoni E., Bergeron R., Gonyou H.W., Brown J.A., Crowe T., Mainau E., Faucitano L. (2014). Water sprinkling market pigs in a stationary trailer. 1. Effects on pig behaviour, gastrointestinal tract temperature and trailer micro-climate. Livest. Sci..

[23] Curtis S.E. (1983). Environmental Management in Animal Agriculture.

[24] Hahn G. (1985). Management and Housing of Farm Animals in Hot Environments. Stress Physiol. Livest..

[25] Hahn G.L., Gaughan J.B., Mader T.L., Eigenberg R.A. (2009). Chapter 5: Thermal indices and their applications for livestock environments. ASABE Monograph No. 25: Livestock Energetics and Thermal Environment Management.

[26] Bridges T.C., Turner L.W., Gates R.S., Overhults D.G. (2003). Assessing the benefits of misting-cooling systems for growing/finishing swine as affected by environment and pig placement date. Appl. Eng. Agric..

[27] Yanagi T., Xin H., Gates R.S. (2002). Optimization of partial surface wetting to cool caged laying hens. Trans. ASAE.

[28] Green A.R. (2013). Establishing Bedding and Boarding Requirements for Finisher Pigs through Scientific Validation—Micro-Study.

[29] McGlone J., Johnson A., Sapkota A., Kephart R. (2014). Temperature and Relative Humidity Inside Trailers During Finishing Pig Loading and Transport in Cold and Mild Weather. Animals.

[30] McGlone J., Sapkota A., Johnson A., Kephart R. (2014). Establishing Trailer Ventilation (Boarding) Requirements for Finishing Pigs during Transport. Animals.

[31] Kephart R., Johnson A., Sapkota A., Stalder K., McGlone J. (2014). Establishing Bedding Requirements on Trailers Transporting Market Weight Pigs in Warm Weather. Animals.

[32] McGlone J., Johnson A., Sapkota A., Kephart R. (2014). Establishing Bedding Requirements during Transport and Monitoring Skin Temperature during Cold and Mild Seasons after Transport for Finishing Pigs. Animals.

[33] Kephart R., Johnson A., Sapkota A., Stalder K., McGlone J. (2014). Establishing Sprinkling Requirements on Trailers Transporting Market Weight Pigs in Warm and Hot Weather. Animals.

[34] McGlone J.J., Johnson A.K. (2012). Establishing Bedding and Boarding Requirements for Finisher Pigs through Scientific Validation—Macro-Study.

[35] SAS Institute, Inc. (2013). SAS/STAT 9.4 User’s Guide.

[36] Green A.R., Gates R.S., Lawrence L.M. (2007). Equine thermoregulatory responses during summertime road transport and stall confinement. Braz. J. Biosyst. Eng..

[37] Heymsfield C. (2018). Computational Fluid Dynamics Model for Air Velocity through a Poultry Transport Trailer in a Holding Shed. Master’s Thesis.

[38] Purswell J.L., Gates R.S., Lawrence L.M., Davis J.D. (2010). Thermal environment in a four-horse slant-load trailer. Trans. ASABE.

[39] Purswell J.L., Davis J.D., Green A.R., Gates R.S., Lawrence L.M., Coleman R.J. (2003). Measuring ventilation in a horse trailer during transport. 2003 ASAE Annual Meeting.

[40] Green A.R. (2003). Measuring Horse Physiological Response to Transport. Master’s Thesis.

[41] Lenkaitis A.C. (2007). Development of a Microenvironment Measurement System for Swine Transport. Master’s Thesis.

